# Author Correction: Tandem gene duplications contributed to high-level azole resistance in a rapidly expanding *Candida tropicalis* population

**DOI:** 10.1038/s41467-024-44825-y

**Published:** 2024-01-18

**Authors:** Xin Fan, Rong-Chen Dai, Shu Zhang, Yuan-Yuan Geng, Mei Kang, Da-Wen Guo, Ya-Ning Mei, Yu-Hong Pan, Zi-Yong Sun, Ying-Chun Xu, Jie Gong, Meng Xiao

**Affiliations:** 1 Department of Infectious Diseases and Clinical Microbiology, Beijing Institute of Respiratory Medicine and Beijing Chao-Yang Hospital, Capital Medical University, Beijing, 100020 China; 2 Beijing Key Laboratory for Mechanisms Research and Precision Diagnosis of Invasive Fungal Diseases, Department of Laboratory Medicine, State Key Laboratory of Complex Severe and Rare Diseases, Peking Union Medical College Hospital, Chinese Academy of Medical Sciences, Beijing, 100730 China; 3 National Key Laboratory of Intelligent Tracking and Forecasting for Infectious Diseases, National Institute for Communicable Disease Control and Prevention, Chinese Center for Disease Control and Prevention, Beijing, 102206 China; 4 Peking University First Hospital - National Institute for Communicable Disease Control and Prevention Joint Laboratory of Pathogenic Fungi, Beijing, 102206 China; 5 Department of Laboratory Medicine, West China Hospital, Sichuan University, Chengdu, 610041 Sichuan China; 6 Department of Clinical Laboratory, First Affiliated Hospital of Harbin Medical University, Harbin, 150001 Heilongjiang China; 7 Department of Clinical Laboratory, Jiangsu Province Hospital, Nanjing, 210029 Jiangsu China; 8 Department of Clinical Laboratory, Fujian Medical University Union Hospital, Fuzhou, 350001 Fujian China; 9 Department of Clinical Laboratory, Tongji Hospital, Tongji Medical College of Huazhong University of Science and Technology, Wuhan, 430030 Hubei China

**Keywords:** Fungal genomics, Genetic variation, Fungal infection

Correction to: *Nature Communications* 10.1038/s41467-023-43380-2, published online 15 December 2023

The order of authors in the original version of this Article was incorrect. The positions of authors Ying-Chun Xu and Jie Gong were switched. This has now been corrected in the PDF and HTML versions of the Article.

