# Supplementary material for: Tandem gene duplications contributed to high-level azole resistance in a rapidly expanding Candida tropicalis population
Source: Nat Commun. 2023 Dec 15;14:8369. doi: 10.1038/s41467-023-43380-2 (PMC10724272; doi:10.1038/s41467-023-43380-2)
Supplement: Supplementary file 1 — Supplementary Information [file 41467_2023_43380_MOESM1_ESM.pdf]

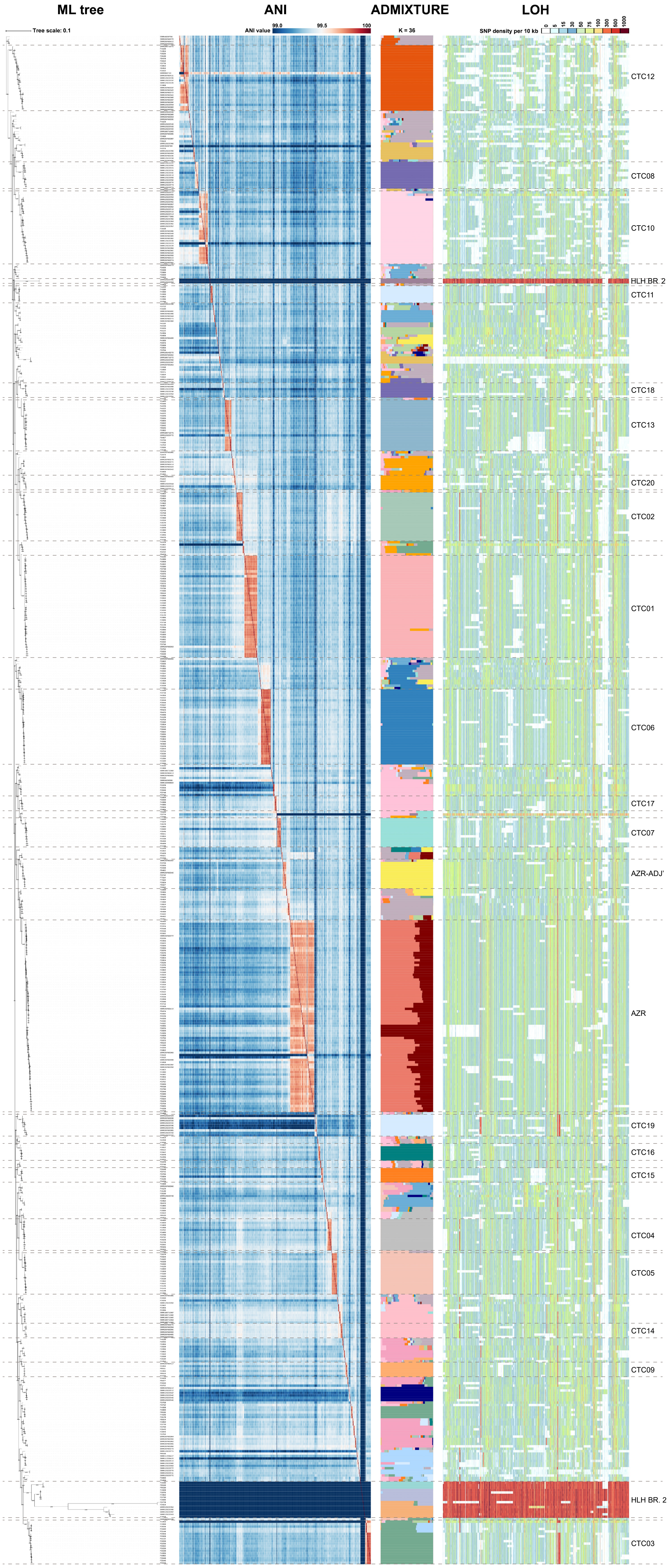

Supplementary Fig. S1. Phylogenetic population structure of 629 *C. tropicalis* isolates based on whole genome sequencing (WGS) results. From left to right: (i) maximum-likelihood (ML) tree based on WGS SNPs; (ii) heatmap of average nucleotide identity analysis (ANI) results; (iv) ADMIXTURE structure analysis results (showing K = 36); (v) loss-of heterozygosity (LOH) analysis (two genome-wide high-level heterozygosity branches [HLH BR.] are shown in dark-red).

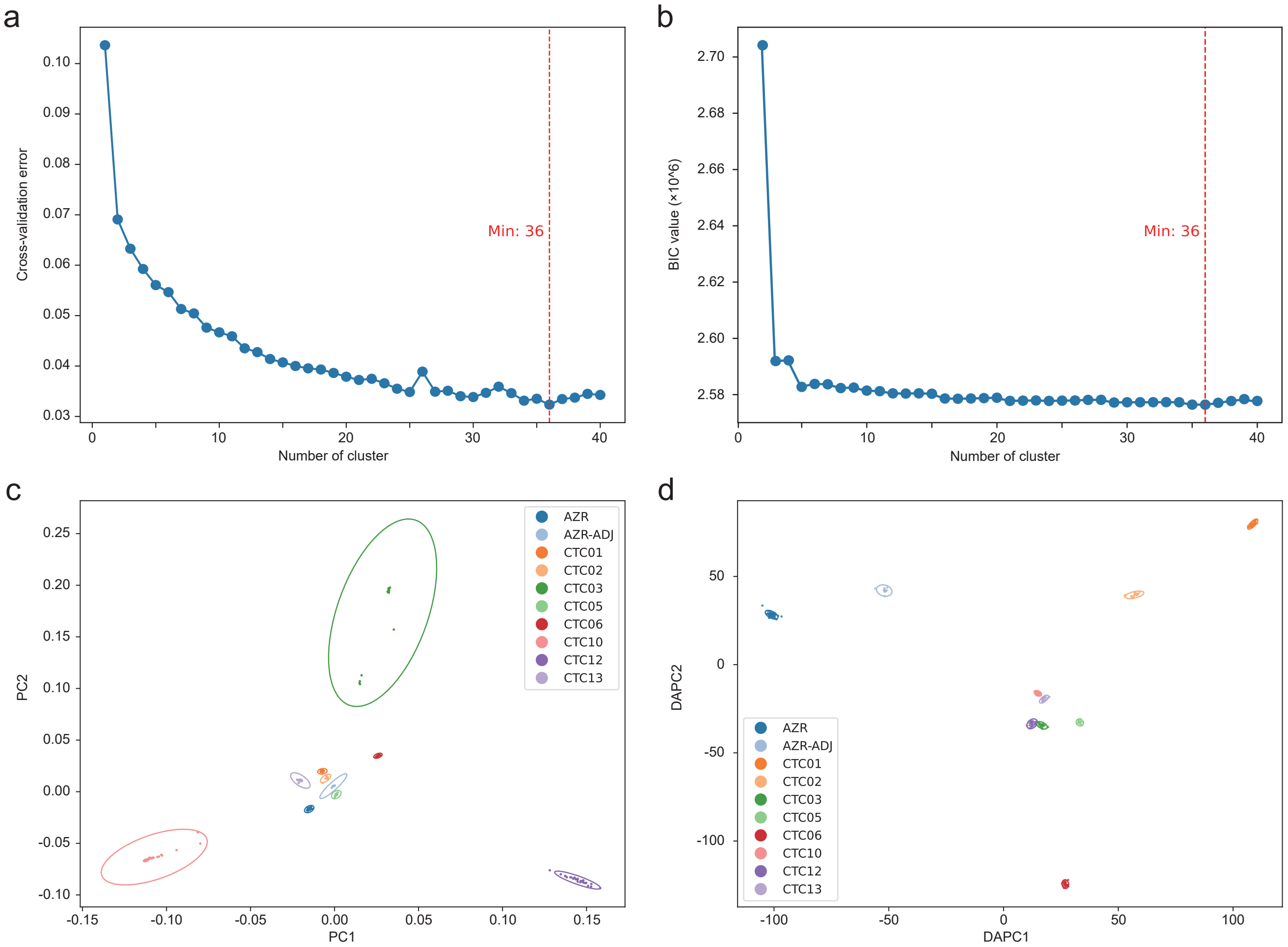

Supplementary Fig. S2. Population genomic analysis for supporting assignment of phylogenetic clusters. a, ADMIXTURE analysis to estimate best number of ancestral populations (K). b, Bayesian Information Criterion (BIC) analysis using a Gaussian Mixture Model (GMM). Both ADMIXTURE cross-validation error and BIC value were minimized at K = 36 (red dashed line). c and d, Principal Component Analysis (PCA, S2c) and Discriminant Analysis of Principal Components (DAPC, S2d) plots showing the ten most prevalent WGS phylogenetic clusters, each distinguished by a unique color. Ellipses around each cluster indicated 95% confidence intervals.
