# Supplementary material for: Tandem gene duplications contributed to high-level azole resistance in a rapidly expanding Candida tropicalis population
Source: Nat Commun. 2023 Dec 15;14:8369. doi: 10.1038/s41467-023-43380-2 (PMC10724272; doi:10.1038/s41467-023-43380-2)
Supplement: Supplementary file 3 — Description of Additional Supplementary Files [file 41467_2023_43380_MOESM3_ESM.pdf]

Description of Additional Supplementary Files

File Name: Supplementary Data 1
Description: Information summary of 1,571 *C. tropicalis* isolates used for MLST analysis in this study.

File Name: Supplementary Data 2
Description: Summary of the 629 *C. tropicalis* isolates used for WGS analysis in this study.

File Name: Supplementary Data 3
Description: Missense mutations and corresponding amino acid substitutions for genes associated with azole resistance in cluster AZR, group AZR-ADJ, and other fluconazole non-susceptible isolates.

File Name: Supplementary Data 4
Description: Unique orthologous groups (OGs) identified in *C. tropicalis* cluster AZR and group AZR-ADJ.

File Name: Supplementary Data 5
Description: Acknowledgments to the principal and co-principal investigators from the hospitals that participated in the CHIF-NET study for their contributions to the isolates collection used in this study.
